# Public awareness of the link between alcohol and cancer in England in 2015: a population-based survey

**DOI:** 10.1186/s12889-016-3855-6

**Published:** 2016-11-30

**Authors:** Penny Buykx, Jessica Li, Lucy Gavens, Lucie Hooper, Melanie Lovatt, Elena Gomes de Matos, Petra Meier, John Holmes

**Affiliations:** 1 School of Health and Related Research (ScHARR), University of Sheffield, Regent Court, 30 Regent Street, Sheffield, S1 4DA UK; 2 Policy Research Centre for Cancer Prevention, Cancer Research UK, Angel Building, 407 St John Street, London, EC1V 4AD UK; 3 Present address: University of Stirling, Stirling, FK94LA UK; 4 Institut für Therapieforschung, Percival Street 25, Munich, 80804 UK

**Keywords:** Alcohol, Cancer, Public, Awareness, Risk factors

## Abstract

**Background:**

Public knowledge of the association between alcohol and cancer is reported to be low. We aimed to provide up-to-date evidence for England regarding awareness of the link between alcohol and different cancers and to determine whether awareness differs by demographic characteristics, alcohol use, and geographic region.

**Methods:**

A representative sample of 2100 adults completed an online survey in July 2015. Respondents were asked to identify which health outcomes, including specific cancers, may be caused by alcohol consumption. Logistic regressions explored whether demographic, alcohol use, and geographic characteristics predicted correctly identifying alcohol-related cancer risk.

**Results:**

Unprompted, 12.9% of respondents identified cancer as a potential health outcome of alcohol consumption. This rose to 47% when prompted (compared to 95% for liver disease and 73% for heart disease). Knowledge of the link between alcohol and specific cancers varied between 18% (breast) and 80% (liver). Respondents identified the following cancers as alcohol-related where no such evidence exists: bladder (54%), brain (32%), ovarian (17%). Significant predictors of awareness of the link between alcohol and cancer were being female, more highly educated, and living in North-East England.

**Conclusion:**

There is generally low awareness of the relationship between alcohol consumption and cancer, particularly breast cancer. Greater awareness of the relationship between alcohol and breast cancer in North-East England, where a mass media campaign highlighted this relationship, suggests that population awareness can be influenced by social marketing.

## Background

Alcohol was classified as a carcinogen by the International Agency for Research into Cancer in 1988 [1] and has been conclusively demonstrated to contribute to the development of cancers of the mouth, throat, oesophagus, breast, liver and bowel [2, 3]. A 2016 evidence review also identified alcohol as a probable cause of stomach cancer [4] and meta-analysis indicating a dose response relationship for prostate cancer is forthcoming [5]. Annually, alcohol accounts for 5.8% of cancer deaths worldwide, [6] while in 2010 in the UK 3.6% of newly diagnosed cancers were attributable to alcohol [7]. However, despite the well-established contribution of alcohol-related cancers to the burden of disease and mortality, literature suggests that public knowledge of the link between alcohol and cancer is poor. In a 2009 UK study, only 14% of people identified alcohol as a risk factor for cancer (unprompted), [8] while a 2014 Australian study found just under half those participating in an online survey selected alcohol among a list of potential cancer risk factors [9]. This is consistent with evidence about public awareness of cancer warning signs, which has also been shown to be low, especially among those who are male, younger people or from lower socio-economic backgrounds [10]. Further, people in more deprived groups are generally diagnosed with cancer at a later stage than those who are less deprived, again indicating that socio-economic gradient may be an important factor to consider in relation to risk factor awareness [11]. Previous research suggests that consideration of differences in cancer awareness by health behaviour is also relevant. For example, Bowden et al. [12] found a significant association between excess alcohol consumption and not perceiving alcohol as an important risk factor for cancer, with higher consumption being associated with lower perception of risk.

Alcohol use is widespread, with approximately four out of five British adults consuming alcohol in 2015 [13]. A recent review of the UK Chief Medical Officers’ guidelines regarding alcohol consumption has resulted in a lowering of the number of alcohol units it is recommended people do not exceed within a given week to 14 units a week for both men and women (1 unit = 7.9 g/10 ml ethanol) [14]. The previous guideline (if the recommended daily limits were multiplied across the week) was 14–21 units/week for women and 21–28 units/week for men. One of the primary justifications given for this reduction was the perceived need for health guidance to reflect the increasing evidence of a dose–response relationship between alcohol and cancer [14]. Updating or developing health promotion material to reflect the revised UK alcohol guidelines would allow governmental organisations and public health advocacy organisations to incorporate information about the carcinogenic potential of alcohol. However, there has been no recent national study on the extent to which the general population are already aware of the relationship between alcohol and cancer against which the impact of such information campaigns could be assessed.

On an international basis, public health advocates, such as cancer prevention charities and alcohol harm reduction organisations, are increasingly interested in raising awareness of the link between alcohol and cancer through social marketing campaigns. For example, in Western Australia in 2011, two such organisations implemented a mass-media campaign (i.e. TV and print advertising, online communication) aimed towards educating women about the increased risk of breast cancer associated with drinking. A recently published evaluation found that over the three waves of the campaign, there was significant improvement in awareness of the increased cancer risk associated with regular drinking [15]. Further, there was an increase in the proportion of people who indicated an intention to reduce drinking, although no change in actual drinking behaviour was detected. In the North East of England, a local public health organisation focussed on reducing alcohol consumption and related harms has recently implemented similar cancer awareness-raising campaigns within their regional footprint [16]. The campaigns have been widely delivered via TV and online, with general cancer awareness campaigns [17] run in Nov-Dec 2013 and Nov-Dec 2014 and breast cancer awareness campaigns [18, 19] run in Nov-Dec 2014 and July 2015. Campaign reach data indicate approximately 59% of adults in the North East (of an adult population of 2.1 million) saw the breast cancer advertisement, on average about eight times [16]. However, as yet, no peer-reviewed evaluations of these campaigns have been published. While awareness-raising campaigns alone may not be sufficient to cause behaviour change - there is as yet little direct evidence for the benefit of mass media campaigns in changing drinking behaviour, although there is strong evidence in relation to tobacco, a field with a larger body of literature [20] – such campaigns can increase understanding of *why* one might consider reducing consumption. Such knowledge is an important factor contributing to behaviour change, according to major behaviour change theories such as COM-B, which posits that individuals require the Capability, Opportunity and Motivation to effect behaviour change (with knowledge regarded as an aspect of Capability) [21].

In the context of changes to UK national guidance regarding alcohol use being in part related to the cancer-causing potential of alcohol and the likelihood of public health campaigns to communicate this, it is of interest to better understand the extent of any knowledge gap in this area. We therefore aimed to provide up-to-date evidence for England regarding public awareness of the link between alcohol and cancer generally, and for different types of cancer (i.e. cancer sites), and to identify population subgroup differences in awareness of the alcohol-cancer link.

## Methods

### Recruitment and response rate

A cross-sectional online survey of alcohol-related health knowledge was conducted in July 2015. A nationally representative sample of 2100 English adults aged 18 and over was recruited by an independent market research company. Volunteer members of an existing market research panel (Vision One) were invited to participate in a survey on ‘health and lifestyle’ behaviours. Of the 11,846 members sent an email invitation, half (n = 5929) clicked the ‘Start your survey’ link and 2480/5929 (41.8%) were deemed eligible to proceed based on quota sampling by sex, age, region and education. Respondents with incomplete or invalid responses were excluded (n = 380), giving a final sample size of 2100 (i.e. 84.7% of those who were eligible and started the survey). The average time for completion of the survey was thirteen minutes (median = 9.9).

### Ethics approval and consent to participate

Ethical approval for the survey was granted by the School of Health and Related Research Ethics Committee, University of Sheffield. Upon opening the survey, panel members were directed to an information page about the study, including contact details for the Ethics Committee. Respondents then provided consent to participate by clicking a link to start the survey.

### Measures

Respondents were asked via an open ended question to identify any health conditions they thought could result from drinking too much alcohol. Irrespective of responses to the open-ended question, respondents were then asked to indicate which of seven listed health conditions they thought could result from drinking too much alcohol: cancer, heart disease, diabetes, high cholesterol, liver disease, being overweight or obese, and arthritis (this question was based on a previous study [9] which did not include arthritis as a response option: this condition was assumed not to be alcohol related and was added to check the discriminant validity of questions). Finally, respondents were asked “Do you think your risk of developing the following types of cancer is increased by drinking alcohol?” followed by a list; stomach, ovarian, breast, mouth & throat, brain, colon & rectal, liver, and bladder cancer. Of these, ovarian, brain and bladder cancer were assumed not to be alcohol-related. Response options were “yes”, “no”, and “don’t know” for all closed questions.

Demographic information available included age in years, gender, highest level of education (no qualifications, below degree level, degree or above), region of residence (nine English regions), and 2015 Index of Multiple Deprivation (IMD) quintile based on postcode [22]. The IMD is an area-based measure of deprivation used in the UK and is calculated on the basis of 37 indicators across seven weighted domains (income – weight = 22.5%, employment - 22.5%, education, skills and training - 13.5%, crime - 9%, barriers to housing and services - 9.3%, and living environment - 9.3%), and resulting IMD scores are assigned to each of 32,844 small areas in England [22, 23]. Alcohol use was measured employing the Alcohol Use Disorders Test short form (AUDIT-C), [24] a widely accepted three item screening tool used to identify higher risk drinking. Scores range from 0 to 12. Scores were dichotomised into <5 (i.e. non or lower risk drinker) and 5+ (higher risk drinker) [25].

### Analysis

A dichotomous *unprompted cancer awareness* variable (yes/no) was created indicating whether individuals reported “cancer” (i.e. where any mention of general or specific cancer was made) when asked about any health conditions that could result from drinking too much in the open ended question. A *prompted cancer awareness* variable was created for those who selected cancer from a list of potentially alcohol-related health conditions. Regarding awareness of the alcohol-related risk of specific cancer types; for those cancers with a known link to alcohol, responses were dichotomised into “yes” and “no or don’t know”, while for those with no known link, responses were dichotomised into “no” and “yes or don’t know”. As the evidence concerning whether or not stomach cancer is alcohol-related was equivocal at the time the survey was conducted, “yes”, “no” and “don’t know” responses were analysed separately.

Statistical analyses were conducted on the complete sample of 2100 respondents. Pearson Chi-square and t-tests were used for bivariate comparisons. Respondents with missing data on IMD (n = 21) were excluded from multivariate analyses. Six logistic regression models were conducted to estimate odds-ratios (OR) and 95% confidence intervals (CI) for 1) unprompted cancer awareness, 2) prompted cancer awareness, and awareness of four alcohol-related cancers 3) liver, 4) breast, 5) colon & rectal and 6) mouth & throat cancers. These models adjusted for gender, education (no qualifications, below degree, degree or above), IMD score quintile (five categories from most deprived to least deprived), region (9 regions total: North East, North West, Yorkshire and the Humber, West Midlands, East Midlands, East of England, London, South East, South West), AUDIT-C score (<5, 5+) and age (entered as a continuous variable in years) using the forced entry (i.e. single step) method in SPSS V.22.0 for Windows. We followed the approach recommended by Hosmer et al. in including all intuitively relevant variables in the multivariate analysis to control for possible confounding between variables [26]. Sample weights were created and used to adjust for the under-sampling of those without qualifications relative to quotas based on population data for England and Wales from the 2011 Census [27].

## Results

### Sample description

Of the 2100 respondents, 51% were female and the mean age was 47.8 (range 18–80, SD = 16.62). Thirty percent had degree or above degree level educational qualifications, 55% had below degree level, and 15% did not have qualifications. The proportion of respondents in each IMD quintile, from most to least deprived, were 22.8, 22.6, 20.3, 16.7 and 16.6% respectively (1% missing data). Two fifths (41.3%) were higher risk drinkers. The number and percentage of survey respondents from each region relative to adult population statistics is presented in Appendix.

### Unprompted and prompted cancer awareness

Unprompted, only 12.9% of respondents identified cancer as a potential consequence of drinking too much alcohol, and when presented as part of a list of health conditions, 46.9% selected cancer, with a further 29.0% indicating “don’t know” and 24.1% “no” (Table 1). Cancer was the least frequently identified of the alcohol-related conditions.

**Table 1:** Proportion of respondents who believe (a) health condition ‘can result from drinking too much alcohol’; (b) risk of specific cancer type is increased by drinking alcohol

(a) General health condition	Believe health condition can result from drinking too much alcohol (*N* = 2100)		
	Yes (%)	No (%)	Don’t know (%)
Alcohol-related conditions			
Liver disease	94.6	2.4	3.0
Being overweight or obese	83.8	7.4	8.7
Heart disease	73.3	10.0	16.7
Diabetes	58.5	15.9	25.6
High cholesterol	52.1	19.7	28.1
Cancer	46.9	24.1	29.0
Condition not related to alcohol			
Arthritis	14.3	46.2	39.5
(b) Specific cancer type	Believe risk of specific cancer type is increased by drinking alcohol (*N* = 2100)		
	Yes (%)	No (%)	Don’t know (%)
Alcohol-related cancers			
Liver	80.0	5.8	14.2
Colon and rectal	38.5	23.0	38.5
Breast	17.8	38.7	43.5
Mouth and throat	48.1	19.5	32.4
Cancer potentially related to alcohol			
Stomach	57.1	13.6	29.3
Cancers not related to alcohol			
Bladder	54.3	15.0	30.7
Brain	31.8	27.2	41.1
Ovarian	16.5	38.0	45.5

When presented with a list of specific cancers, the proportion of respondents who correctly identified alcohol as a causative factor for cancer varied by cancer type. For those cancer types for which alcohol is a known risk factor; awareness ranged from 17.8% for breast cancer up to 80.0% for liver cancer (Table 1). Substantial uncertainty was also apparent, with 14.2% saying “don’t know” for liver cancer and between 29.3 and 45.5% saying “don’t know” for the other alcohol-related cancer types. For three cancer types where alcohol is not a recognised risk factor, correct knowledge ranged from 15.0% for bladder cancer to 38.0% for ovarian cancer. At the time the survey was conducted evidence for whether or not stomach cancer is alcohol-related stomach cancer was equivocal and so we have not identified a ‘correct’ answer for this question: 57.1% of respondents endorsed it as alcohol related.

### Bivariate predictors of cancer awareness

For both the unprompted and prompted questions about cancer awareness, bivariate comparisons revealed being female, more highly educated, and region of residence were all significantly associated with both unprompted and prompted cancer awareness, while younger age was associated with prompted awareness, but not unprompted (Table 2). There was a large difference by education level in prompted cancer awareness, while the effect for gender was modest. Descriptively, a quarter (25.2%) of respondents in the North East region mentioned cancer unprompted, compared to 11-13% elsewhere; and 64% when prompted, compared to 43-51% elsewhere. There were no significant differences in unprompted and promoted awareness by IMD quintile or AUDIT-C category at the bivariate level.

**Table 2:** Bivariate associations with awareness that cancer ‘can result from drinking too much alcohol’ (*N* = 2100)

Characteristic	Number	Unprompted cancer awareness			Prompted cancer awareness		
		Cancer mention	No cancer mention	*P* ^a^	Yes	No/Don’t know	*P* ^a^
		%	%		%	%	
Overall	2100	12.9	87.1	-	46.9	53.1	-
Gender				0.020			0.006
Male	1030	11.2	88.8		43.9	56.1	
Female	1070	14.6	85.4		49.8	50.2	
Education				0.026			<0.001
No qualifications	315	9.5	90.5		33.7	66.3	
Below degree	1155	12.5	87.5		47.3	52.7	
Degree or above	630	15.6	84.4		52.9	47.1	
IMD quintile^c^				0.230			0.164
5 Most deprived	479	12.7	87.3		43.0	57.0	
4	474	10.1	89.9		45.7	54.3	
3	426	13.8	86.2		48.1	51.9	
2	351	15.4	84.6		48.9	51.1	
1 Least deprived	349	13.5	86.5		51.1	48.9	
Region				0.025			0.016
North East	115	25.2	74.8		63.5	36.5	
North West	320	11.6	88.4		45.5	54.5	
Yorkshire & The Humber	207	13.0	87.0		44.2	55.8	
East Midlands	182	10.4	89.6		45.1	54.9	
West Midlands	218	11.9	88.1		42.9	57.1	
East of England	186	11.8	88.2		44.1	55.9	
London	285	12.3	87.7		44.8	55.2	
South East	378	13.2	86.8		50.9	49.1	
South West	209	12.4	87.6		46.9	53.1	
Audit C				0.276			0.118
< 5	1234	12.2	87.8		45.5	54.5	
5+	866	13.9	86.1		49.0	51.0	
		Mean (SD)	Mean (SD)	*P* ^b^	Mean (SD)	Mean (SD)	*P* ^b^
Age	2100	47.7 (16.7)	47.9 (16.6)	0.852	46.8 (16.4)	48.8 (16.8)	0.007

^a^Based on χ2 test

^b^Based on *t*-test

^c^Missing cases (*n* = 21) are not presented here

Bivariate comparisons for each specific cancer type showed greater awareness among females (except for colon & rectal cancer) and those who were more highly educated (except for breast cancer), as well as significant regional differences for breast cancer awareness only (Table 3). Almost 30% of respondents from the North East of England correctly identified breast cancer as alcohol-related compared to less than 20% in all other regions. There were no differences in awareness that liver, breast, colon & rectal and mouth & throat cancer are alcohol-related by IMD quintile, AUDIT-C category, or age.

**Table 3:** Bivariate associations with awareness that the risk of liver, breast, colon & rectal, and mouth & throat cancer is increased by drinking alcohol (*N* = 2100)

	Number	Liver			Breast			Colon and rectal			Mouth and throat		
		Yes %	No/DK %	*P* ^a^	Yes %	No/DK %	*P* ^a^	Yes	No/DK %	*P* ^a^	Yes	No/DK %	*P* ^a^
Overall	2100	80.0	20.0	-	17.8	82.2	-	38.5	61.5	-	48.1	51.9	-
Gender				<0.001			0.014			0.190			0.020
Male	1030	76.3	23.7		15.7	84.3		37.1	62.9		45.5	54.5	
Female	1070	83.6	16.4		19.8	80.2		39.9	60.1		50.6	49.4	
Education				<0.001			0.281			0.002			<0.001
No qualifications	315	73.0	27.0		16.8	83.2		30.5	69.5		39.4	60.6	
Below degree	1155	79.6	20.4		17.0	83.0		38.6	61.4		46.8	53.2	
Degree or above	630	84.3	15.7		19.8	80.2		42.4	57.6		54.8	45.2	
IMD quintile^c^				0.317			0.773			0.862			0.056
5 Most deprived	479	78.1	21.9		19.6	80.4		37.0	63.0		43.0	57.0	
4	474	80.8	19.2		16.7	83.3		38.9	61.1		47.8	52.2	
3	426	81.5	18.5		17.1	82.9		39.6	60.4		50.2	49.8	
2	351	77.7	22.3		18.3	81.7		40.0	60.0		47.4	52.6	
1 Least deprived	349	82.8	17.2		17.2	82.8		37.2	62.8		53.0	47.0	
Region				0.943			0.033			0.344			0.750
North East	115	80.0	20.0		29.6	70.4		44.3	55.7		53.0	47.0	
North West	320	79.0	21.0		17.9	82.1		34.8	65.2		48.4	51.6	
Yorkshire & The Humber	207	79.8	20.2		14.4	85.6		40.4	59.6		44.7	55.3	
East Midlands	182	81.9	18.1		18.1	81.9		39.6	60.4		47.8	52.2	
West Midlands	218	80.2	19.8		18.0	82.0		37.3	62.7		46.1	53.9	
East of England	186	79.0	21.0		13.4	86.6		32.3	67.7		46.8	53.2	
London	285	78.7	21.3		19.9	80.1		37.9	62.1		45.3	54.7	
South East	378	79.8	20.2		17.5	82.5		40.6	59.4		50.7	49.3	
South West	209	83.7	16.3		15.3	84.7		42.1	57.9		50.7	49.3	
Audit C				0.059			0.284			0.828			0.291
< 5	1234	81.4	18.6		17.1	82.9		38.3	61.7		47.1	52.9	
5+	866	78.1	21.9		18.9	81.1		38.8	61.2		49.4	50.6	
		Mean (SD)	Mean (SD)	*P* ^b^	Mean (SD)	Mean (SD)	*P* ^b^	Mean (SD)	Mean (SD)	*P* ^b^	Mean (SD)	Mean (SD)	*P* ^b^
Age	2100	47.7 (16.8)	48.6 (15.8)	0.323	47.0 (15.9)	48.0 (16.8)	0.282	48.5 (16.0)	47.4 (17.0)	0.143	47.2 (16.7)	48.5 (16.5)	0.076

*DK* Don’t know

^a^Based on *χ*2 test

^b^Based on *t*-test

^c^Missing cases (*n* = 21) are not presented here

There were no significant differences in correctly identifying bladder cancer as unrelated to alcohol use by any of the predictor variables (Table 4). There was a difference in awareness that brain cancer is not alcohol-related by AUDIT-C category and age, with a greater proportion of higher risk drinkers and older respondents aware of this. Awareness that ovarian cancer is not alcohol related was significantly higher among women, the more highly educated, higher risk drinkers, and older respondents.

**Table 4:** Awareness that the risk of bladder, brain, or ovarian cancer is not increased by drinking alcohol; perception of whether stomach cancer alcohol-related (*N* = 2100)

	Number	Bladder			Brain			Ovarian			Stomach			
		No %	Yes/DK %	*P* ^a^	No %	Yes/DK %	*P* ^a^	No %	Yes/DK %		No %	DK %	Yes %	*P* ^a^
Overall	2100	15.0	85.0	-	27.2	72.8	-	38.0	62.0	-	13.6	29.3	57.1	
Gender				0.392			0.107			0.013				0.001
Male	1030	15.6	84.4		28.7	71.3		35.3	64.7		16.3	29.0	54.7	
Female	1070	14.3	85.7		25.6	74.4		40.6	59.4		10.9	29.7	59.4	
Education				0.893			0.115			0.002				<0.001
No qualifications	315	15.9	84.1		23.2	76.8		29.8	70.2		15.2	38.1	46.7	
Below degree	1155	14.9	85.1		27.0	73.0		38.2	61.8		14.3	28.1	57.6	
Degree or above	630	14.8	85.2		29.5	70.5		41.7	58.3		11.6	27.0	61.4	
IMD quintile^d^				0.772			0.099			0.469				0.702
5 Most deprived	479	13.8	86.2		27.3	72.7		38.2	61.8		13.8	28.8	57.4	
4	474	16.5	83.5		29.7	70.3		39.9	60.1		15.6	27.2	57.2	
3	426	14.8	85.2		29.5	70.5		39.7	60.3		13.4	29.1	57.5	
2	351	15.4	84.6		25.7	74.3		36.0	64.0		12.6	32.7	54.7	
1 Least deprived	349	13.8	86.2		22.0	78.0		34.6	65.4		11.5	30.1	58.5	
Region				0.179			0.643			0.259				0.056
North East	115	13.8	86.2		21.7	78.3		27.8	72.2		9.6	25.2	65.2	
North West	320	11.9	88.1		28.5	71.5		37.2	62.8		11.6	30.7	57.7	
Yorkshire & The Humber	207	15.5	84.5		27.9	72.1		37.5	62.5		11.5	34.1	54.3	
East Midlands	182	19.8	80.2		28.0	72.0		38.5	61.5		17.0	23.6	59.3	
West Midlands	218	16.5	83.5		26.7	73.3		41.5	58.5		15.6	29.8	54.6	
East of England	186	14.0	86.0		25.3	74.7		34.4	65.6		13.4	36.6	50.0	
London	285	14.3	85.7		25.3	74.7		37.9	62.1		14.7	30.1	55.2	
South East	378	17.8	82.2		30.8	69.2		42.1	57.9		16.4	26.8	56.8	
South West	209	11.0	89.0		24.9	75.1		36.8	63.2		9.1	26.4	64.4	
Audit C				0.054			0.001			0.048				0.010
< 5	1234	13.7	86.3		24.6	75.4		36.2	63.8		12.1	31.3	56.6	
5+	866	16.7	83.3		30.8	69.2		40.5	59.5		15.8	26.5	57.7	
		Mean (SD)	Mean (SD)	*P* ^b^	Mean (SD)	Mean (SD)	*P* ^b^	Mean (SD)	Mean (SD)	*P* ^b^	Mean (SD)	Mean (SD)	Mean (SD)	*P* ^c^
Age	2100	46.4 (16.8)	48.1 (16.6)	0.094	46.1 (16.4)	48.5 (16.7)	0.004	45.9 (16.9)	49.0 (16.4)	<0.001	47.1 (16.7)	49.5 (16.2)	47.2 (16.8)	0.014

*DK* Don’t know

^a^Based on *χ*2 test

^b^Based on *t*-test

^c^Based on one-way ANOVA

^d^Missing cases (*n* = 21) are not presented here

### Multivariate predictors of cancer awareness

Logistic regression results (Table 5) for unprompted cancer awareness and prompted cancer awareness, revealed that awareness was predicted by being female, being more highly educated and living in the North East region (with those from the North East being between 2.4 and 3.0 times more likely to mention cancer unprompted than those from any other region). Deprivation quintile, AUDIT-C category and age were not associated with unprompted or prompted cancer awareness. Awareness of four alcohol-related cancer sites (liver, breast, colon & rectal, and mouth & throat) was also associated with being more highly educated (except breast cancer) and being female (except colon & rectal cancer). However, in contrast to the results for cancer awareness more generally, living in the North East was only predictive of knowing that breast cancer, but not three other cancers, is alcohol-related, with those from the North East being between 1.8 and 2.7 times as likely to be aware of this link as those from any other region. Those in the most deprived quintile were less likely to know that mouth & throat cancers are associated with alcohol compared to the least deprived. There was a significant association detected between being older and knowing that colon & rectal cancer is alcohol related, although the odds ratio was small.

**Table 5:** Multivariate logistic regression results for factors associated with cancer awareness (*N* = 2079)

	Unprompted cancer awareness		Prompted cancer awareness		Liver		Breast		Colon & Rectal		Mouth & Throat	
	OR	95% CI	OR	95% CI	OR	95% CI	OR	95% CI	OR	95% CI	OR	95% CI
Gender												
Male	1.00		1.00		1.00		1.00		1.00		1.00	
Female	1.42*	(1.09-1.86)	1.27*	(1.06-1.52)	1.49*	(1.19-1.86)	1.41*	(1.12-1.79)	1.13	(0.94-1.36)	1.23*	(1.03-1.47)
Education												
No qualifications	1.00		1.00		1.00		1.00		1.00		1.00	
Below degree	1.28	(0.85-1.97)	1.56*	(1.19-2.06)	1.50*	(1.10-2.04)	0.91	(0.64-1.30)	1.56*	(1.18-2.07)	1.23	(0.94-1.60)
Degree or above	1.69*	(1.06-2.69)	1.96**	(1.44-2.65)	2.13**	(1.48-3.06)	1.11	(0.76-1.64)	1.90**	(1.39-2.60)	1.66*	(1.23-2.24)
IMD quintile^†^												
5 (Most deprived)	0.98	(0.63-1.51)	0.77	(0.57-1.03)	0.84	(0.57-1.22)	1.11	(0.75-1.62)	1.16	(0.86-1.58)	0.71*	(0.53-0.96)
4	0.73	(0.47-1.13)	0.84	(0.63-1.12)	0.94	(0.65-1.37)	0.92	(0.63-1.35)	1.16	(0.87-1.56)	0.86	(0.65-1.15)
3	1.07	(0.71-1.64)	0.92	(0.69-1.23)	0.93	(0.63-1.36)	0.98	(0.67-1.44)	1.18	(0.87-1.58)	0.93	(0.70-1.24)
2	1.23	(0.80-1.89)	0.97	(0.72-1.32)	0.75	(0.51-1.09)	1.12	(0.76-1.67)	1.18	(0.87-1.61)	0.85	(0.63-1.14)
1 (Least deprived)	1.00		1.00		1.00		1.00		1.00		1.00	
Region												
North East	1.00		1.00		1.00		1.00		1.00		1.00	
North West	0.37**	(0.21-0.64)	0.47*	(0.30-0.74)	0.92	(0.54-1.57)	0.49*	(0.30-0.80)	0.69	(0.44-1.06)	0.81	(0.53-1.25)
Yorkshire Humber	0.42*	(0.23-0.76)	0.45*	(0.28-0.73)	0.97	(0.54-1.73)	0.39*	(0.22-0.67)	0.87	(0.55-1.40)	0.69	(0.44-1.10)
East Midlands	0.33*	(0.17-0.62)	0.44*	(0.27-0.72)	1.02	(0.56-1.85)	0.52*	(0.30-0.90)	0.82	(0.51-1.32)	0.78	(0.48-1.25)
West Midlands	0.37*	(0.20-0.67)	0.43**	(0.27-0.69)	1.01	(0.57-1.79)	0.51*	(0.30-0.87)	0.77	(0.49-1.23)	0.74	(0.47-1.17)
East of England	0.37*	(0.20-0.69)	0.44*	(0.27-0.72)	0.90	(0.50-1.62)	0.36*	(0.20-0.65)	0.61	(0.38-1.00)	0.75	(0.46-1.20)
London	0.41*	(0.23-0.71)	0.43**	(0.27-0.68)	0.86	(0.50-1.50)	0.57*	(0.35-0.95)	0.78	(0.50-1.22)	0.70	(0.45-1.09)
South East	0.41*	(0.24-0.69)	0.55*	(0.35-0.85)	0.90	(0.53-1.54)	0.49*	(0.30-0.80)	0.90	(0.58-1.38)	0.84	(0.55-1.29)
South West	0.36*	(0.19-0.66)	0.47*	(0.29-0.76)	1.26	(0.66-2.16)	0.41*	(0.23-0.72)	0.93	(0.58-1.49)	0.86	(0.54-1.36)
AUDIT C												
< 5	0.88	(0.67-1.16)	0.93	(0.77-1.12)	1.26	1.00-1.58	0.86	(0.68-1.09)	0.99	(0.82-1.19)	0.94	(0.78-1.13)
5+	1.00		1.00		1.00		1.00		1.00		1.00	
Age per year of increase	1.00	(0.99-1.01)	1.00	(0.99-1.00)	1.00	(0.99-1.01)	1.00	(0.99-1.01)	1.01*	(1.00-1.01)	1.00	(0.99-1.00)

*OR* Odds ratios; 95% CI = 95% confidence interval

^†^Missing cases (*n* = 21) are not presented here *p < 0.05; **p < 0.001

## Discussion

### General and specific alcohol-related cancer awareness

Our findings highlight a continued lack of public understanding of the carcinogenic nature of alcohol. For most people, cancer was not ‘top of mind’ when asked about the potential health consequences of alcohol use, with only about one in eight people mentioning cancer, suggesting there has not been any appreciable improvement in levels of awareness among the English population since a 2009 study found 14% of respondents identified cancer as a potential outcome of alcohol consumption [8]. Even when prompted, only one in two people in our study recognised the link between alcohol and cancer in general and, when specific cancer types were considered, only one in five people were aware of the link to breast cancer, two in five were aware of the link to colon and rectal cancer and one in two of the link to mouth and throat cancer. Further, irrespective of whether actually alcohol-related or not, a substantial minority of people selected “don’t know” for each of the cancers included in this study (with the exception of liver cancer) and more people thought some cancers for which there is no evidence of alcohol-relatedness (e.g. bladder, brain) were linked to alcohol than correctly identified breast cancer as alcohol-related, both of which underscore our finding of generally low awareness. It is of interest to note that the most frequently identified alcohol-related cancer, liver cancer, is one of the least frequently occurring in the UK, whilst breast cancer, for which there was poor awareness is one of the most frequent [7]. It is possible that public health alcohol reduction messages may be perceived as less personally relevant if people are only aware of the carcinogenic potential of alcohol in relation to less common cancers rather than the range of cancer types potentially caused.

### Characteristics associated with awareness

In the context of emerging evidence of increased risk of cancer at even low levels of consumption, [2] publication of revised drinking guidelines for England, and the emphasis placed on the carcinogenic potential of alcohol by the Chief Medical Officers and their Guideline Development Group in setting the new, lower threshold, [14] there is potentially the impetus for those organisations involved in the development and dissemination of alcohol and/or cancer public health messages to undertake awareness raising campaigns. In addition to revealing an overall low level of awareness of the link between alcohol and cancer, as discussed above, our findings could inform awareness raising efforts by identifying in which groups of the population, and for which cancer types, knowledge is the lowest. For example, there is some evidence to support targeting information to those with lower levels of education. Only about a third of those without educational qualifications identified alcohol as a cancer risk when prompted compared to half of those with a university degree. This group was also significantly less likely to be aware of the alcohol cancer link on all outcome variables except breast cancer, for which knowledge was uniformly low. While the explanatory pathway for the relationship between education and health knowledge is as yet unclear (i.e. while these factors are known to correlate, the relationship is not necessarily directly causal), [28] our analysis has revealed those with low levels of education as a priority audience. Promisingly, however, we found no difference in current levels of awareness by social gradient, as measured by IMD. As the World Health Organisation recommends that the equity effects of universal public health interventions be evaluated, [29] any efforts to improve public awareness should be monitored to determine whether knowledge gains are made equally across all socio-economic strata.

The finding that men are marginally (although statistically significantly) less likely than women to be aware of the link between alcohol and cancer suggests that information dissemination strategies should be designed to reach both male and female audiences. While alcohol consumption has risen among women in recent decades and there is increasing evidence about the risk of breast cancer, [30, 31] alcohol consumption among men is still on average higher than that among women, [32] as are rates of harm [13]. We found no difference between non- or low risk drinkers and higher risk drinkers in levels of awareness on any of the outcome variables. Therefore, even if social marketing campaigns were to specifically target those who drink, or who drink at higher risk levels, the levels of awareness revealed by this study can be regarded as a baseline indication of knowledge in 2015 across the whole population regardless of drinking level, against which future improvements could be monitored.

### Regional differences

Our findings are strongly indicative that the social marketing efforts to improve alcohol-related cancer awareness in the North East region of England have had at least a short term effect on awareness. Although our study only measured awareness at a single point in time, data collection coincided with the July 2015 wave of an alcohol and breast cancer awareness campaign, [19] in which a 40 s TV advertisement was shown repeatedly and supplemented with news media exposure and online promotion [16]. This campaign followed an earlier alcohol and breast cancer awareness campaign run in Nov-Dec 2014 using a static image (news media and online only) [18] and a general cancer awareness campaign run in Nov-Dec 2013 and Nov-Dec 2014 (TV advertisement, news media and online [17]. These campaigns were confined to the North East region, [16] and so far as we are aware, no other regions of England have been exposed to a comparable population-level mass media campaign. While a pre-post study design including measures of campaign exposure would provide more robust effectiveness evidence, the strong inter-regional and breast cancer-specific effects lend weight to the hypothesis that the campaigning undertaken in the North East has contributed to the higher levels of awareness in that region. Although not part of our original analysis plan, given these findings and the sex-specific nature of breast cancer, we subsequently examined the level of unprompted, prompted, and breast cancer awareness in the North East region by gender. Awareness among women in the North East on these measures was 27.5, 72.5 and 41.2% respectively compared with 23.1, 56.3 and 20.0% for men in the North East (and 12.9, 46.9 and 17.8% for the sample as a whole). This suggests that while awareness on all three measures was higher in the North East (for both women and men) than elsewhere, future research should examine gender effects following exposure to cancer awareness measures.

### Methodological considerations

An analytical consideration for our study was the decision to include three predictor variables which are related to one another; education to IMD, and IMD to region. However, all three of these potential predictors were of interest in their own right: level of formal education has been shown to be correlated to health knowledge, [28] there are known social inequalities in cancer outcomes, [33] and the known regional differences in recent cancer awareness social marketing practices provided a rationale for examining current levels of awareness by region. For these reasons all three variables were included independently. A further analytical consideration is that the number of bivariate tests conducted increased the risk of a Type 1 error. We considered performing a correction such as Bonferroni, but were conscious of the increased possibility of a Type 2 error associated with such an approach, [34] and as all variables were to be subsequently included in logistic regression analyses, made no adjustment for multiple testing. We instead caution the reader to be aware of the increased risk of a chance finding within the bivariate analysis.

A potential limitation of our study was our sample was recruited from a volunteer online market research panel and it is not clear to what extent this may have biased prevalence estimates. However, quota sampling ensured respondents were representative of the general population of England with respect to age, gender, region, and education level. The response rate was also satisfactory for survey research. As mentioned above, the causal inferences than can be drawn between the campaign run in the North East of England and the higher levels of awareness in that region are limited due to the cross sectional nature of this research.

### Future research directions

It is not clear to what extent participant ‘awareness’ represented certain knowledge of the link between alcohol and cancer. Future research could explore level of participant certainty in their responses. Also of interest for future research is the pattern of results in relation to which cancers were and were not believed to be related to alcohol. Perceptions of (any) cancer risk have been described as an ‘embodied’ phenomenon [35]. In our study, the majority of people incorrectly selected bladder cancer as alcohol-related, suggesting that they perceive there may be a carcinogenic effect of alcohol associated with the urinary tract. Similarly, a third incorrectly selected brain cancer, which given that excessive alcohol use can often result in a headache and/or forgetfulness, would seem a reasonable choice in the absence of certain knowledge. In contrast, the act of alcohol consumption has no obvious physical link to the development of breast cancer, a site far from the gastro-intestinal tract through which alcohol passes. Future qualitative work could explore perceptions of embodied risk and the biological mechanisms by which cancers develop, and the extent to which these may contribute to misunderstanding which of the cancers are associated with lifestyle factors, particularly alcohol. Such research would shed light on whether providing an explanation of causal mechanisms could be a useful avenue to explore in the development of future public health campaigns. As noted in the introduction, improved awareness of the health harms associated with alcohol alone will not necessarily prompt individual behaviour change. Recent Australian research has found that only about half those exposed to alcohol health warning labels including a cancer message thought this would influence drinking behaviour, however, the majority thought it would prompt conversations about the cancer risk associated with alcohol use [36]. A useful future direction for research in this area would be to analyse the content of publically available discussion fora on this issue (e.g. public submissions regarding the new drinking guidelines, commentary on media articles) to consider not only how increased awareness is incorporated into drinkers’ existing knowledge and views on alcohol and health, but also how it might shape individual drinking behaviour and impact on public and political attitudes towards other structural interventions intended to reduce alcohol related harms.

## Conclusion

There is generally low awareness of the relationship between alcohol consumption and cancer, including for specific cancer types. Awareness of the link between alcohol consumption and breast cancer is particularly low. Greater awareness of the relationship between alcohol and breast cancer in North East England, where a recent mass media campaign highlighted this relationship, suggests that population awareness can be influenced by social marketing.

## Appendix

**Table 6:** Total population aged 18+ of English regions in mid-2015 and proportional representation of each region in the overall survey sample

English region	Regional population aged 18 + ^a^	% total population aged 18+	Survey sample	% total sample (*n* = 2100)
North East	2,100,204	4.9	115	5.5
North West	5,652,470	13.1	320	15.2
Yorkshire and The Humber	4,244,933	9.8	207	9.9
East Midlands	3,705,500	8.6	182	8.7
West Midlands	4,489,117	10.4	218	10.4
East	4,776,467	11.1	186	8.9
London	6,720,843	15.6	285	13.6
South East	7,029,838	16.3	378	18.0
South West	4,389,099	10.2	209	10.0
Total	43,108,471	100.0	2100	100.0

^a^ Office for National Statistics (2016) *Population Estimates for UK, England and Wales, Scotland and Northern Ireland.* https://www.ons.gov.uk/peoplepopulationandcommunity/populationandmigration/populationestimates/datasets/populationestimatesforukenglandandwalesscotlandandnorthernireland
